# Multimodality Imaging in the Detection of Ischemic Heart Disease in Women

**DOI:** 10.3390/jcdd9100350

**Published:** 2022-10-13

**Authors:** Sean Paul Gaine, Garima Sharma, Albree Tower-Rader, Mina Botros, Lara Kovell, Anushri Parakh, Malissa J. Wood, Colleen M. Harrington

**Affiliations:** 1 Department of Medicine, Johns Hopkins University School of Medicine, Baltimore, MD 21287, USA; 2 Ciccarone Center for the Prevention of Cardiovascular Disease, Johns Hopkins University School of Medicine, Baltimore, MD 21287, USA; 3 Department of Radiology, Massachusetts General Hospital, Boston, MA 02114, USA; 4 Division of Cardiology, Massachusetts General Hospital, Boston, MA 02214, USA; 5 Department of Medicine, University of Massachusetts Medical School, Worcester, MA 01605, USA; 6 Corrigan Women’s Heart Health Program, Massachusetts General Hospital, Boston, MA 02214, USA

**Keywords:** ischemic heart disease, noninvasive imaging, stress testing cardiovascular disease in women

## Abstract

Women with coronary artery disease tend to have a worse short and long-term prognosis relative to men and the incidence of atherosclerotic cardiovascular disease is increasing. Women are less likely to present with classic anginal symptoms when compared with men and more likely to be misdiagnosed. Several non-invasive imaging modalities are available for diagnosing ischemic heart disease in women and many of these modalities can also assist with prognostication and help to guide management. Selection of the optimal imaging modality to evaluate women with possible ischemic heart disease is a scenario which clinicians often encounter. Earlier modalities such as exercise treadmill testing demonstrate significant performance variation in men and women, while newer modalities such as coronary CT angiography, myocardial perfusion imaging and cardiac magnetic resonance imaging are highly specific and sensitive for the detection of ischemia and coronary artery disease with greater parity between sexes. Individual factors, availability, diagnostic performance, and female-specific considerations such as pregnancy status may influence the decision to select one modality over another. Emerging techniques such as strain rate imaging, CT-myocardial perfusion imaging and cardiac magnetic resonance imaging present additional options for diagnosing ischemia and coronary microvascular dysfunction.

## 1. Introduction

### Background

Cardiovascular disease is the leading cause of death and disability, accounting for approximately one in three deaths worldwide [1]. It is the leading cause of death in the United States; coronary artery disease (CAD) and ischemic heart disease (IHD) remain the leading causes of death attributable to cardiovascular disease in men and women [2]. The incidence of atherosclerotic cardiovascular disease is increasing due to an increase in cardiac risk factors such as obesity, diabetes, and hypercholesterolemia in addition to an aging population [3]. However, age-adjusted mortality rates are decreasing, likely due to improved medical therapies allowing patients to live longer with IHD [4]. Although cardiovascular disease is more common in men, women with CAD have a worse short and long-term prognosis [5]. In addition, women with symptoms of angina or who have had an abnormal cardiac stress test are less likely to be referred for additional diagnostic testing and initiated on guideline directed medical therapies [6,7].

## 2. Discussion

### 2.1. Etiologies

The pathogenesis of atherosclerosis begins in adolescence and early adulthood in the form of fatty streaks, long before manifesting as clinically overt disease [8]. Lipid-rich atherosclerotic plaque may ulcerate or rupture causing thrombosis and ischemia. Atherosclerosis resulting in plaque rupture or erosion is by far the most common cause of myocardial infarction (MI) in men and women.

MI with nonobstructive coronary arteries (MINOCA) accounts for 5–10% of MI. Similarly, myocardial ischemia may occur with signs and symptoms of ischemia in the absence of significant CAD which is classified as ischemia with no obstructive coronary artery disease (INOCA) [9].

There is likely some degree of overlap between MINOCA and INOCA. MINOCA is defined by criteria for the universal definition of MI, in which there is rise and/or fall of cardiac troponin (cTn) value above the 99th percentile with signs and symptoms of myocardial ischemia. This can be manifested as new ischemic ECG changes, new ischemic wall motion abnormalities, the loss of viable myocardium on noninvasive imaging, or identification of coronary thrombus by angiography or autopsy without obstructive coronary artery stenosis (<50% stenosis) on angiography, and the absence of myocarditis or Takotsubo cardiomyopathy [10]. In contrast to acute MI due to CAD, MINOCA commonly affects younger patients, particularly women with fewer traditional cardiac risk factors. The etiology of this condition is heterogenous and may result from plaque disruption by rupture or erosion, coronary vasospasm, spontaneous coronary artery dissection (SCAD), coronary artery embolization or coronary microvascular disease. However, in a majority of cases no etiology is identified [11]. MINOCA can also present with cardiac arrest and heart failure and while the mortality rate tends to be lower than MI due to CAD overall, outcomes are significantly worse than age-matched controls compared with MI secondary to CAD [12].

INOCA is characterized by the presence of angina with non-obstructive CAD (where obstructive CAD is defined as >50–70% stenosis or fractional flow reserve (FFR) <0.8) on angiography. Approximately 60% of patients with INOCA are women. INOCA may occur alone or in combination with hypertension, severe aortic stenosis, severe anemia, coronary vasospasm, and myocardial bridging [13]. In the WISE study, which studied clinically stable women with signs and symptoms of myocardial ischemia, women with INOCA had an elevated risk of all-cause mortality (13 vs. 2.8%) as compared with a representative cohort of women who were matched for age, gender and observed over a similar time period [14]. The concept of plaque vulnerability is an important consideration in these patients whereby non-obstructive plaque with high-risk histological features such as a large necrotic core, thin fibrous cap (<65 mm), active inflammation, angiogenesis, plaque hemorrhage, positive remodeling and microcalcification confer an increased risk of erosion or rupture. Some of these features are detectable by non-invasive imaging techniques such as CCTA, nuclear molecular imaging and CMR. CCTA detection of high-risk plaque is associated with increased risk of future MACE (major adverse cardiovascular outcomes) and may be useful as an additional risk stratification tool, particularly in patients with nonobstructive coronary artery disease, women, and younger patients [15].

Coronary microvascular dysfunction (CMD) is another etiology of IHD in women and is characterized by epicardial, microvascular endothelial or non-endothelial dysfunction. This leads to decreased coronary perfusion which can be detected as reduced coronary flow reserve (CFR). CFR is defined as the maximal increase in flow through the coronary arteries above the normal resting volume which reflects the ability of the coronary circulation to respond to increased oxygen demand. Positron emission tomography (PET) imaging remains the standard method for estimating CFR, although single-photon emission computed tomography (SPECT), magnetic resonance imaging (MRI), intravascular Doppler ultrasound and echocardiography have been used [16]. A ratio of <2.0 at maximal hyperemia compared to baseline capacity is considered abnormal [17]. It is important to note that despite having non-obstructive CAD, patients with CMD are still at an elevated risk for major adverse cardiovascular events [18].

Spontaneous coronary artery dissection (SCAD) results from the separation of the intima and media due to hematoma formation within the medial layer causing external compression of the true coronary arterial lumen resulting in myocardial ischemia and/or infarction. SCAD is likely caused by a combination of various genetic, hormonal, arteriopathy and environmental factors. It is commonly associated with fibromuscular dysplasia which is predominantly seen in female patients. A small minority of cases are attributable to inherited connective tissue disorders such as Marfan, Loeys-Dietz and Ehlers-Danlos [19]. SCAD demonstrates a female predominance, occurring in 87–95% of women [20].

Women with IHD can present differently when compared to men. Women are less likely to present with classic chest pain (31% compared with 42% in men) and more often report dyspnea, weakness, back pain, palpitations, or loss of appetite. Importantly, patients with ACS who present without chest pain are more likely to be misdiagnosed and have a higher risk of death than those who present with classic chest pain symptoms. Moreover, the absence of chest pain has not been consistently correlated with ACS severity [21,22].

### 2.2. Noninvasive Imaging Modalities

**Figure 1:**
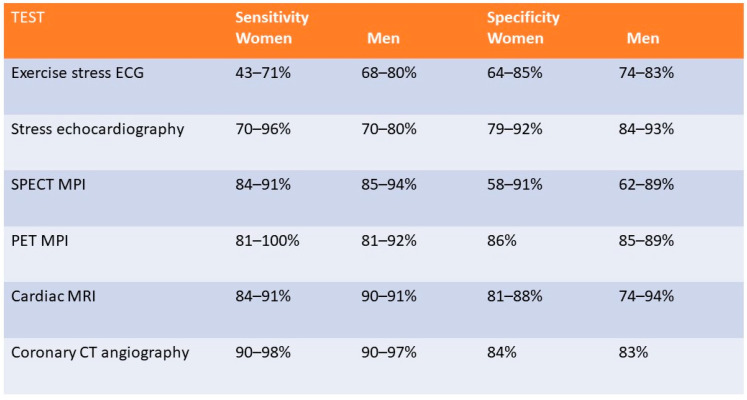
Figure comparing the sensitivity and specificity of different non-invasive imaging modalities in men and women.

There are a variety of different non-invasive imaging modalities that play a role in the diagnosis, management, and prognostic assessment of women with IHD. Overall risk assessment and an understanding of the sex-specific differences in the presentation and pathophysiology of ischemia and performance of available diagnostics is imperative when deciding the optimal test for evaluating women for IHD. Female-specific considerations such as exposure of breast tissue to radiation and pregnancy status necessitate a patient-centered approach to diagnosis. Compared with men, women less frequently have obstructive coronary artery disease or undergo coronary angiography and are more likely to have coronary microvascular dysfunction [7,23]. Figure 1 compares the sensitivity and specificity for the available diagnostic noninvasive modalities for the detection of IHD in men and women.

### 2.3. Exercise Stress Testing

Exercise stress test electrocardiography (ETT) evaluates for inducible ischemia by the presence or absence of ST-segment changes (ST depressions or elevations) along with the assessment of hemodynamic response to exercise and overall cardiorespiratory fitness. Exercise is performed on a treadmill or bicycle ergometry with continuous ECG monitoring. This modality is widely accessible and inexpensive [24]. However, the sensitivity and specificity to detect obstructive CAD is significantly lower in women, as shown in Figure 1 [25,26,27,28,29]. A meta-analysis of the detection of obstructive CAD using ETT, demonstrated a sensitivity of 61% and specificity of 70% in women compared to 68% and 77%, respectively, in men [26]. However, the ROMICAT study (Rule Out Myocardial Infarction Using Computer-Assisted Tomography) which examined 220 patients who underwent ETT demonstrated a sensitivity of 60% for >50% stenosis and a specificity of 94% with a negative predictive value of 78% in women, which was similar to 81% observed in men [30]. The utility of ETT as an initial diagnostic strategy in low-risk, exercising women has been reinforced by results from the What Is the Optimal Method for Ischemia Evaluation in Women (WOMEN) trial, where ETT was shown to have similar 2-year outcomes while providing significant cost-savings [31].

### 2.4. Exercise and Dobutamine Stress Echocardiography

Stress echocardiography allows for the visualization of abnormal myocardial contractility during exercise when compared to baseline, an indication of inducible myocardial ischemia. Exercise is preferred; however, a pharmacological stress agent such as dobutamine can be used to detect new or worsening left ventricular (LV) regional wall motion abnormalities (RWMA). In addition to the qualitative assessment of myocardial response to stress, quantitative measurements with techniques such as strain rate imaging (SRI) derived from tissue Doppler imaging (TDI) can be employed to assess for myocardial viability. Strain describes the shortening, thickening, and lengthening of the myocardium, which can be used as a measure of regional left ventricular function on echocardiography and can detect characteristic findings suggestive of ischemic myocardium such as reduced peak systolic strain, systolic lengthening, and post-systolic shortening [32]. This results in a contraction variable which is independent of the passive tethering effects from other regions that can influence the interpretation of single point velocities measured by TDI and has been successfully used to differentiate between different myocardial viability states [33]. Stress echocardiography is widely available and avoids exposure to ionizing radiation. Contraindications include uncontrolled hypertension, severe arrhythmias, significant left ventricular outflow tract obstruction, ACS, and symptomatic severe aortic stenosis [34]. Overall, stress echocardiography has a higher sensitivity and specificity than ETT in women with a sensitivity of 70 to 96% and specificity 79 to 92% [35,36]. The performance of stress echocardiography to detect inducible ischemia in men and women has been variable, with some studies showing statistical differences [37,38] while other studies did not show significant variations in performance [38,39]. A recent trial that included approximately 45% women observed a sensitivity of 95.4% and a specificity of 96% for the identification of obstructive CAD [40].

### 2.5. SPECT- Myocardial Perfusion Imaging (Exercise and Pharmacological)

SPECT-myocardial perfusion imaging (MPI) provides information about global and regional LV systolic contractility, LV volume, as well as myocardial perfusion defects during exercise or pharmacological stress for the evaluation of patients with known or suspected CAD. Radionucleotide tracers, most commonly 99m-technetium (Tc99m)-labeled perfusion agents are injected intravenously at peak exercise or following vasodilator administration (agents such as adenosine, dipyridamole or regadenoson) or dobutamine administration. Both rest and stress images are typically obtained; however, in selected patients a stress-only protocol may be employed with rest images obtained only if stress images are abnormal [41]. This modality demonstrates good performance in women, with a sensitivity reported of 84–91% and a specificity of 58–91% [42,43,44,45]. SPECT-MPI can also be used to risk stratify patients by assessing the extent and severity of defect size and its degree of reversibility [46,47]. However, limitations in women include false positive results from breast attenuation and reduced accuracy in patients with smaller sized hearts, which is more commonly seen in women [48]. Additional consideration should also be given to radiation exposure, estimated at 11 mSV in this modality, particularly in younger patients. However, current estimates of radiation risk do not demonstrate a differential risk from SPECT-MPI in women relative to men [49,50].

### 2.6. Stress Positron Emission Tomography

**Figure 2:**
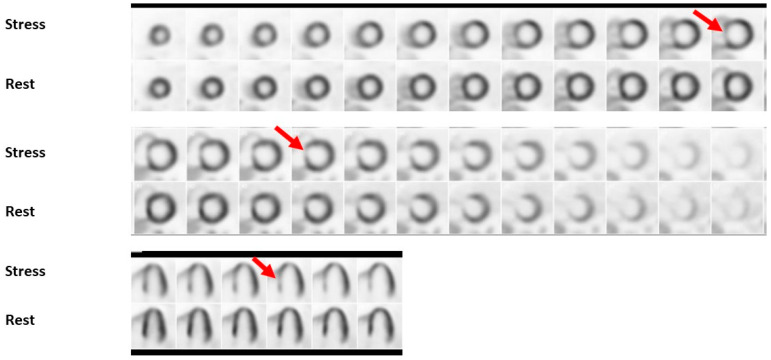
Shows a regadenoson myocardial perfusion PET/CT study using 13-N ammonia tracer. A large perfusion defect of moderate intensity primarily involving the mid-basal septal LV segments with extension to the mid-basal anterior LV segments, which appeared completely reversible, is visible (red arrows). There was also a medium-sized, mild perfusion defect involving the true LV apex and apical LV segments, which appeared reversible.

**Figure 3:**
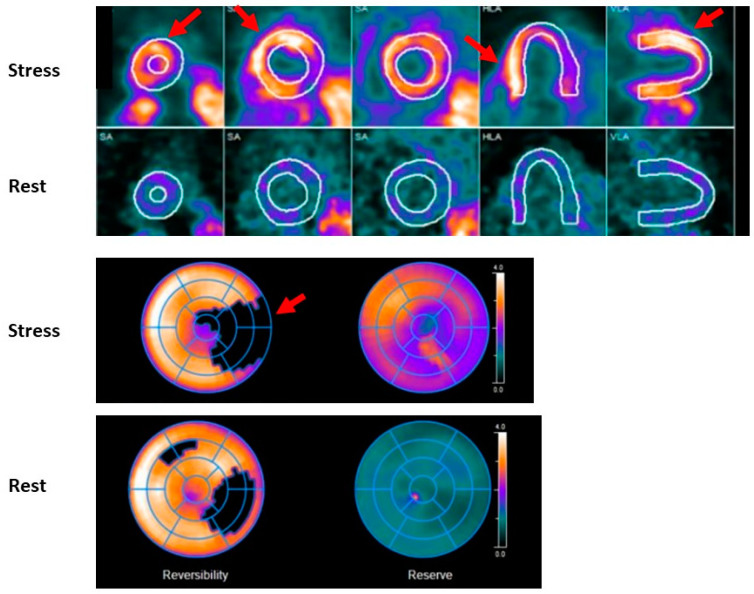
Shows a regadenoson myocardial perfusion PET study. A large, mostly fixed, defect of severe intensity involving the entire inferolateral wall and basal anterolateral wall, as well as the apex is visible (red arrows). Minimal reversibility was noted in the apical lateral wall.

Stress myocardial PET imaging provides similar information to SPECT-MPI imaging while providing information about CFR; which can aid in the diagnosis of CMD; and quantifying global and regional myocardial blood flow (MBF). Quantification of MBF requires accurate measurement of the total tracer activity transported by arterial blood and delivered to the myocardium over a certain period of time. Time-activity curves are acquired (arterial isotope activity versus time) using image regions located in the arterial pool (LV; left atrium or aorta) [51]. The test is also performed in a similar manner whereby rest CT and PET images are attained using radiotracers; either rubidium-82 or 13N-ammonia; which are injected intravenously. These tracers have a very short half-life; therefore; stress images are obtained within 30–80 min following rest images depending on the washout period of the tracer used (see Figure 2 and Figure 3). As a result; pharmacological stress is typically performed due to their short duration of action [52]. The use of PET imaging relative to SPECT is primarily limited by availability. This modality has been shown to be superior to SPECT with respect to image quality; interpretive certainty; and diagnostic accuracy in both men and women [53]. Radiation dose is significantly less than SPECT imaging; estimated at 2–3 mSV depending on the tracer with a similar sensitivity and specificity in men and women as shown in Figure 1 [54,55]. A large meta-analysis including 1442 patients reported an overall sensitivity of 92% and specificity of 85% [56]. A study of 409 patients underwent a baseline PET scan which was repeated 2–3 years later to assess the clinical outcomes of pharmacologic therapy and lifestyle modifications as well as their impact on noninvasive imaging. Patients were sorted into three categories: “poor” treatment without diet or lipid-lowering drugs; or who were actively smoking; “moderate” treatment on American Heart Association diet and lipid-lowering drugs or on strict low-fat diet without lipid-lowering drugs; and “maximal” treatment with strict low-fat diet; regular exercise; and lipid-active drugs dosed to specific target goals of low-density lipoproteins; high-density lipoproteins and triglycerides. Over five years; coronary events occurred in 6.6%, 20.3%, and 30.6% of patients on maximal; moderate; and poor treatment; respectively (*p* = 0.001). Size and/or severity of perfusion abnormalities significantly decreased for patients receiving maximal treatment and increased for patients undergoing moderate and poor treatment (*p* = 0.003 and 0.0001; respectively) [57] Nuclear imaging may also be used detect inflammation within plaque using specific molecular targets. PET-CT imaging using radiotracers such as 18F-fluorodeoxyglucose (18F-FDG) and 68Gallium (68Ga-DOTATATE) allows for combined anatomic identification of coronary plaques coupled with molecular inflammation which has been as-sociated with atherosclerotic plaque progression [58,59]. Increased coronary 18F-fluoride (18F-NaF) uptake has been associated with more rapid progression of coronary calcification and has been used to identify and localize ruptured and high-risk coronary plaque [60,61]. Feasibly their use could permit the identification of patients at increased risk of adverse events or determine which lesions are vulnerable and may benefit from revascularization.

### 2.7. Coronary CT Angiography

**Figure 4:**
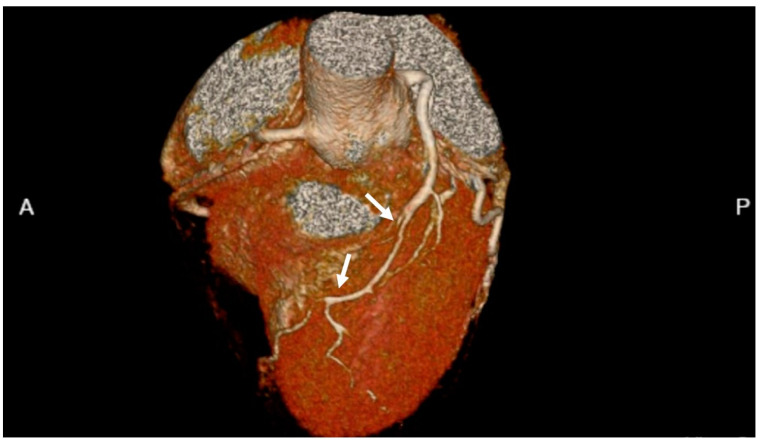
Shows a CCTA 3-D reconstruction with a long segment type II coronary artery dissection in the mid-to-distal left anterior descending coronary artery (white arrows indicating area of dissection). A is anterior, P is posterior.

**Figure 5:**
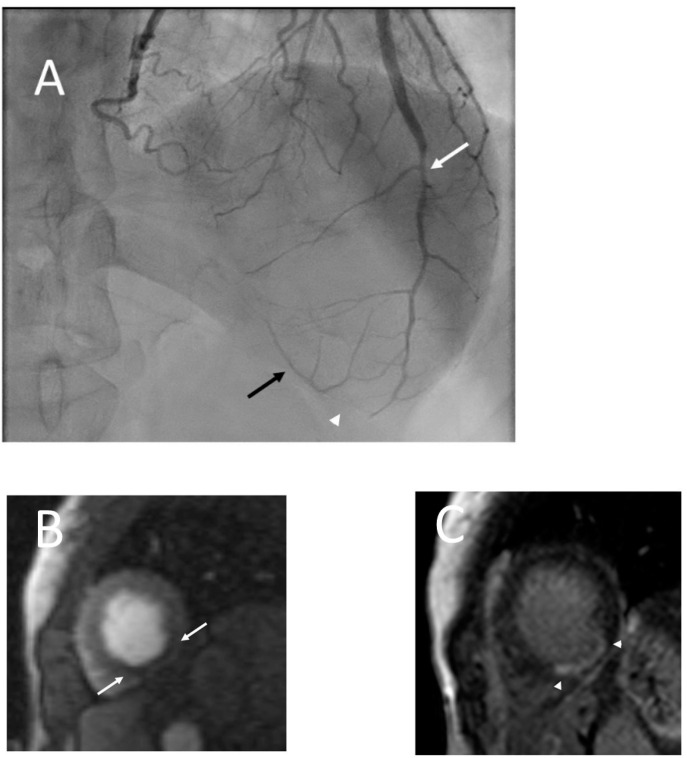

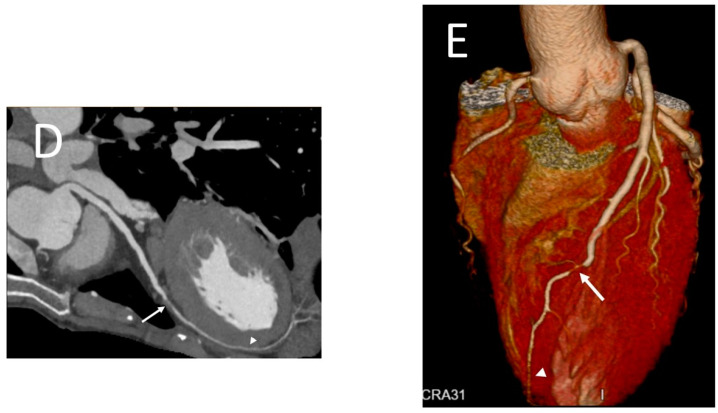
Series shows catheter angiogram (**A**) which shows moderate stenosis of the mid left anterior descending coronary artery (LAD; white arrow) and occlusion of the distal LAD (white arrowhead) with distal collaterals (black arrow). No other significant stenosis in rest of the coronary arteries (not shown) seen, findings which are consistent with SCAD. Cardiac MR (**B**,**C**) performed later revealed resting perfusion defect (**B**) in the apical inferior wall (arrow) with corresponding subendocardial (near 75%) late gadolinium enhancement (arrowhead; **C**) consistent with an infarct in the distribution of the left anterior descending coronary artery. Three days after discharge and cardiac MR, the patient presented again with band-like c lower chest pain and coronary CT angiogram (**D**,**E**) was performed. Curved planar reformat (**D**) and volume rendered (**E**) images now revealed focal severe stenosis (white arrows) in the mid LAD with recanalization of the distal segment (arrowhead) compared to the catheter angiogram performed five days prior.

**Figure 6:**
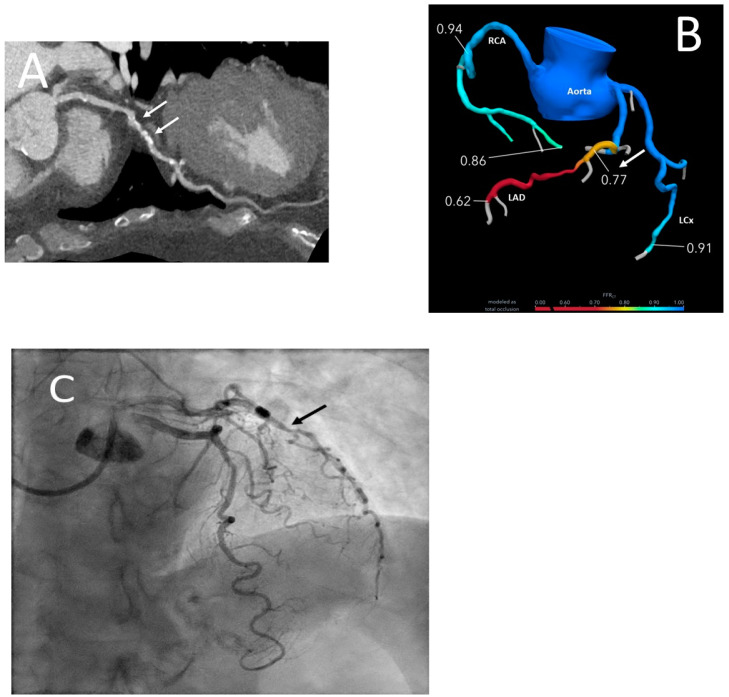
Series shows a CT coronary angiogram with curved planar reformat image (**A**) demonstrating moderate narrowing (arrow) of the proximal-mid left anterior descending coronary artery (LAD). Subsequent CT-fractional flow reserve (CT-FFR; **B**) which revealed hemodynamically significant stenosis with a value of 0.77 distal to the area of concern on CT angiogram (>0.80 is considered negative). Catheter angiogram (**C**) showing presence of moderate stenosis in the proximal-mid LAD (black arrow).

Coronary CT angiography (CCTA) provides detailed information of coronary anatomy with regard to the severity and extent of obstructive and non-obstructive atherosclerosis, plaque location, and characterization. It can also be a useful noninvasive imaging modality for the diagnosis of SCAD (Figure 4 and Figure 5D,E) [62]. This test is widely available and performed using gated-CT scanning with IV iodinated contrast [63]. While coronary CT has very high diagnostic accuracy with excellent spatial and temporal resolution, image quality may be reduced in patients with higher heart rates, morbid obesity, dense coronary calcifications and in those who have undergone previous percutaneous coronary artery interventions with coronary stenting. Furthermore, the use of potentially nephrotoxic iodinated contrast media may limit its use in patients with chronic kidney disease [64]. Nonetheless, CCTA has very high overall sensitivity and specificity for obstructive CAD, 90–95% and 79–95%, respectively [65,66,67]. CCTA can also detect certain vulnerable plaque characteristics such as positive remodeling or low attenuation plaque. These have been associated with poorer outcomes both in obstructive and non-obstructive CAD [68]. Some studies have found that CCTA may be more accurate than noninvasive functional testing for the detection of significant CAD when compared to invasive angiography. However, the landmark PROMISE (Prospective Multicenter Imaging Study for Evaluation of Chest Pain) did not show evidence of superiority relative to functional testing overall [69,70]. Additionally, sex-specific analyses of the ACCURACY trial (Assessment by Coronary Computed Tomographic Angiography of Individuals Undergoing Invasive Coronary Angiography) showed equivalent sensitivity (98% in women versus 97% in men) and specificity (84% in women versus 83% in men) for the detection of CAD [71]. Likewise, the Multi Center Combined Non-invasive Coronary Angiography and Myocardial Perfusion Imaging Using 320-Detector Computed Tomography (CORE-320) trials, which combined the use of CCTA and SPECT, demonstrated similar performance with some incremental benefits with the addition of CT myocardial perfusion imaging (CT-MPI) in women which was not evident in men. CT-MPI is an emerging modality which combines anatomic imaging with additional functional information by detecting pharmacologic hyperemia from the myocardial enhancement patterns after injection of contrast medium to quantify MBF. This has advantages in patients with dense calcifications or in whom the functional impact of a stenotic lesion is uncertain, its widespread use has been limited, however, due to its relative complexity and increased radiation exposure [72]. CT-fractional flow reserve (CT-FFR) is a more available, emerging modality which can reduce the false positive rate associated with CCTA by reclassifying lesions by their hemodynamic significance. CT-FFR uses CCTA technology to create a patient-specific geometric model of the coronary arteries and applies mathematical models to simulate pharmacological stress across a stenotic segment (Figure 6B) [73]. It has been shown to increase specificity relative to standard CCTA and help guide management, and it has high diagnostic accuracy which has been validated against invasive angiography and pressure wire assessment [74,75,76]. Sex-related differences in CT-FFR exist and women have been found to have higher CT-derived FFR measurements for the same degree of stenosis relative to men. Women with positive CT-derived FFR also tend to have less obstructive CAD at invasive angiography with less revascularization [77]. However, despite these differences, a CT-FFR-guided revascularization strategy appears equally beneficial among men and women and it has shown similar diagnostic accuracy and discriminatory power for ischemia detection between sexes [78,79].

Although typically diagnosed by invasive angiography, CCTA may also be of value in the diagnosis and follow-up of SCAD, which affects women in most cases. Typical features of SCAD on CCTA include abrupt luminal stenosis, intramural hematoma, tapered luminal stenosis, dissection and arterial tortuosity [80]. A normal CT study does not rule out SCAD however and detection of SCAD by CT has its limitations. Involved distal vessels may be beyond scanner resolution or intramural hematoma may be misidentified as artifact or non-calcified atherosclerotic plaque [81].

CCTA can provide prognostic data for all-cause mortality and IHD events. Analyses based on the CONFIRM (Coronary CT Angiography Evaluation For Clinical Outcomes: An International Multicenter) registry showed a progressive increase in mortality with the extent and severity of CAD (adjusted hazard ratios of 1.6, 2.0, 2.9, and 3.7, respectively, for nonobstructive, 1-vessel, 2-vessel, and 3-vessel obstructive CAD compared with no CAD) [82].

Concerns regarding exposure to ionizing radiation with CCTA, specifically to breast tissue in women, have been appropriately raised. However, subgroup analyses of the PROMISE trial compared CCTA and functional testing with regard to safety, incidental findings and effective radiation dose in 9470 patients. CCTA had a similar 90-day cumulative radiation dose compared to functional testing. However, in the subgroup whose physicians intended nuclear stress (CTA 3147; nuclear 3203), CTA had lower median index test (8.8 vs. 12.6 mSv, *p* < 0.001) and 90-day cumulative (11.6 vs. 13.1 mSv, *p* < 0.001) doses, independent of patient characteristics. The lowest nuclear doses employed 1-day Tc-99m protocols (12.2 mSv). The lowest CTA doses were at sites performing ≥500 CTAs/year (6.9 mSv) and with advanced (latest available) CT scanners (5.5 mSv) [83]. Breast displacement for CCTA has been shown to lower the overall radiation dose received (mean of 2.3 mSv) in women who achieved 1/3 less radiation exposure compared with BMI-matched male counterparts in one study [84].

### 2.8. Stress Cardiac Magnetic Resonance Imaging

Stress CMR can provide accurate assessment of myocardial ischemia, viability, and function. The absence of ionizing radiation with CMR combined with its high contrast and spatial resolution are advantageous, especially in younger or pregnant women. Stress CMR images are obtained with the use of vasodilators (typically adenosine, regadenoson or dipyridamole) to induce hyperemia followed by a gadolinium-based contrast agent injected peripherally; serial T1-weighted CMR images are then acquired. The contrast enters normally perfused myocardial regions more quickly and in higher concentrations which can be detected as a greater increase in T1-signal relative to abnormally perfused regions [85]. Although not widely available, CMR has the potential to quantify MBF and detect CMD defined by invasive coronary reactivity testing [86]. Limitations of stress CMR include limited availability and the inability to include an exercise component. Additionally, there is concern for nephrogenic systemic fibrosis in patients with advanced CKD; however, currently used agents have a low estimated risk (<0.07%) of this complication [87]. Finally, patient tolerability and implanted ferromagnetic devices or objects may preclude its use in certain patients.

Stress CMR has high diagnostic accuracy owing to its high spatial resolution, ability to differentiate and characterize the layers of the myocardium, and lack of limitation by attenuation artifacts. This high diagnostic accuracy was shown in the MR-IMPACT II (Magnetic Resonance Imaging for Myocardial Perfusion Assessment in Coronary Artery Disease Trial) and CE-MARC (Clinical Evaluation of Magnetic Resonance Imaging in Coronary Heart Disease) trials which enrolled patients with suspected angina to stress CMR and SPECT imaging and compared them to invasive coronary angiography [88]. The CE-MARC trial showed that CMR was more sensitive 87% versus 67%, respectively, and similarly specific 83% when compared with SPECT [89]. Subsequent analyses using CE-MARC trial data demonstrated that, unlike SPECT, there were no significant differences in diagnostic performance of CMR between men and women [90]. Other studies analyzing CMR performance in men and women have shown that CMR has high diagnostic specificity among women, approximately 84–91%, which is comparable to that seen in men; there is also similar sensitivity for the detection of obstructive CAD [91,92]. One large recent meta-analysis reported sensitivity as high as 90% and specificity of 94% [93]. CMR can also provide prognostic and risk-stratification information. In a study evaluating 405 patients, 168 of whom were women, ischemia demonstrated by CMR showed a strong association with major adverse cardiovascular events (15%) when compared with 0.3% in patients without evidence of ischemia, regardless of patient sex [94]. CMR is also very useful in MINOCA [95]. The etiology of this condition is heterogenous and can be considered a working diagnosis while multiple potential causes are evaluated [96]. CMR has the ability to detect MINOCA mimics such as myocarditis and Takotsubo cardiomyopathy, as well as hypertrophic and dilated cardiomyopathy [97]. Moreover, CMR can detect specific infarct patterns which can help differentiate the etiology of myocardial injury with transmural or subendocardial patterns typical of ischemic injury, subepicardial or mid-wall typical of non-ischemic injury, and multiple sub-endocardial hyper-enhanced small areas corresponding to the same vascular territory typical of embolic injury (Figure 5B,C) [98,99].

### 2.9. Safety of Non-Invasive Imaging Modalities for Ischemia in Women and Pregnancy

**Figure 7:**
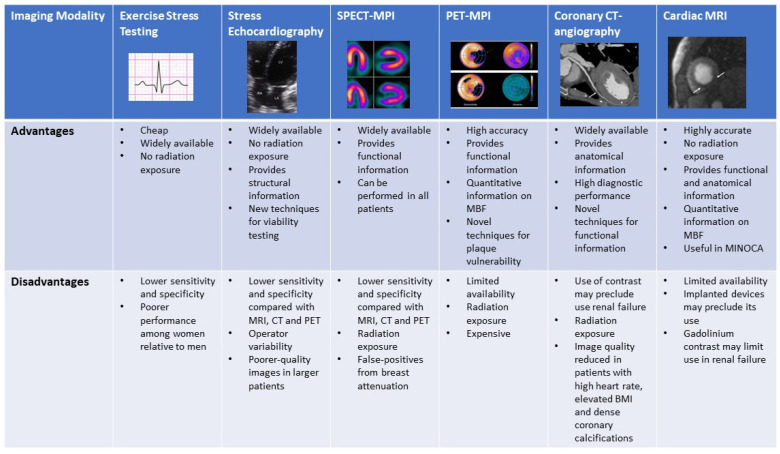
Figure comparing the advantages and disadvantages of the various non-invasive imaging modalities.

A range of effective, non-invasive modalities are available for evaluation of ischemia in women. Exposure of breast tissue to radiation and pregnancy or breastfeeding status are important female-specific considerations which can influence selection. Figure 7 compares the advantages and disadvantages of each modality.

Exercise stress ECG testing can be considered an option in all women regardless of pregnancy status given its safety profile [100]. Exercise stress echocardiography is similarly safe for use in pregnancy and avoids exposure to ionizing radiation. Pharmacological stress echocardiography also appears to be similarly safe in pregnancy with no significant maternal or fetal risk; dobutamine and dipyridamole are considered category B agents favored over adenosine, which is category C [101].

PET and SPECT-MPI imaging involve radiation exposure and radiotracer use which are generally contraindicated in pregnancy. The society for nuclear medicine also advises against MPI in women who are breastfeeding but notes that, for those who do receive 99mTc or 13N-ammonia, no interruption to breastfeeding is necessary [102]. The radiation dose varies from 3–11 mSv for SPECT and 2–3 mSv for PET depending on the protocol used. CCTA similarly exposes patients to ionizing radiation; however, more advanced scanners and techniques to reduce radiation exposure, such as breast displacement, have resulted in doses ranging from 2 to 5 mSv [84]. Overall estimates of cancer risk for MPI and CCTA testing is in the range of 3 to 8 per 10,000 tested [36]. Notably, radiation exposure through CT scan or nuclear medicine imaging techniques is at a dose much lower than the exposure associated with fetal harm from a single study [103]. Although iodinated contrast use in CCTA is generally considered safe during pregnancy and lactation, it has been rarely associated with neonatal hypothyroidism [104]. It is important to note that radiation exposure is not an absolute contraindication to CT imaging or nuclear medicine imaging in pregnancy but requires higher justification for use in non-emergent settings.

CMR benefits from lack of radiation exposure and is safe for use in pregnancy, including with the use of gadolinium contrast agents. MRI imaging has not been associated with any mutagenic or teratogenic effects [105]. This modality is preferred in younger or pregnant patients in whom radiation exposure and lifetime risk of cancer warrants additional consideration.

## 3. Conclusions

IHD is the leading cause of morbidity and mortality among women. Women with IHD are more likely to present without classic anginal symptoms, face greater diagnostic delay, poorer prognosis and are less likely to be initiated on guideline-directed pharmacotherapy relative to men. INOCA, CMD and SCAD all more frequently affect women compared with men. A variety of noninvasive imaging modalities are available or are emerging for the detection of IHD in women. Figure 7 provides an overview of the advantages and disadvantages of these different imaging modalities. Although disparities still exist, newer modalities are very specific and sensitive for the detection of ischemia and CAD and have less gender-specific performance variation when compared with the traditionally recommended ETT. No single modality should be chosen in all circumstances. Rather, being cognizant of the differences in the presentation and pathophysiology of ischemia in women, and using a patient-centered approach with careful risk stratification, is key to selecting the optimal diagnostic strategy. Female-specific considerations such as radiation exposure to breast tissue and pregnancy status also warrant consideration. The identification of IHD with or without obstructive CAD is of major importance for appropriate risk stratification and prognostication; women identified with such should be treated with aggressive guideline-directed therapies. Emerging modalities and techniques such as SRI, CT-MPI and CMR present additional options for detecting myocardial ischemia and microvascular dysfunction and their role should continue to be investigated.

## Data Availability

Not applicable.
